# Low-Cost Optical Sensors for Soil Composition Monitoring

**DOI:** 10.3390/s24041140

**Published:** 2024-02-09

**Authors:** Francisco Javier Diaz, Ali Ahmad, Lorena Parra, Sandra Sendra, Jaime Lloret

**Affiliations:** Instituto de Investigación para la Gestión Integrada de Zonas Costeras, Universitat Politècnica de València, Gandía C/Paranimf, 1, 46730 Grao de Gandia, Spain; fjdiabla@doctor.upv.es (F.J.D.); aahmad1@upv.es (A.A.); sansenco@upv.es (S.S.); jlloret@dcom.upv.es (J.L.)

**Keywords:** soil fertilizer, dryland agriculture, WSN, agricultural practices, soil properties, salinity, optical sensor

## Abstract

Studying soil composition is vital for agricultural and edaphology disciplines. Presently, colorimetry serves as a prevalent method for the on-site visual examination of soil characteristics. However, this technique necessitates the laboratory-based analysis of extracted soil fragments by skilled personnel, leading to substantial time and resource consumption. Contrastingly, sensor techniques effectively gather environmental data, though they mostly lack in situ studies. Despite this, sensors offer substantial on-site data generation potential in a non-invasive manner and can be included in wireless sensor networks. Therefore, the aim of the paper is to develop a low-cost red, green, and blue (RGB)-based sensor system capable of detecting changes in the composition of the soil. The proposed sensor system was found to be effective when the sample materials, including salt, sand, and nitro phosphate, were determined under eight different RGB lights. Statistical analyses showed that each material could be classified with significant differences based on specific light variations. The results from a discriminant analysis documented the 100% prediction accuracy of the system. In order to use the minimum number of colors, all the possible color combinations were evaluated. Consequently, a combination of six colors for salt and nitro phosphate successfully classified the materials, whereas all the eight colors were found to be effective for classifying sand samples. The proposed low-cost RGB sensor system provides an economically viable and easily accessible solution for soil classification.

## 1. Introduction

Dryland farming is the practice of growing crops solely with natural rainfall. It involves cultivating crops exclusively through natural precipitation, abstaining from irrigation. It represents a form of sustenance agriculture in regions where inadequate soil moisture impedes crop growth. Sparse and uncertain rainfall, alongside unreliable irrigation setups, delineate arid regions [1]. Dryland farming restricts crop growth to certain periods because of insufficient moisture [2]. The main worries concerning arid regions and soil deterioration revolve around farm output [3] and water accessibility [4,5,6]. Unstable crop yields, recurring droughts, unpredictable weather patterns, substantial soil erosion, deforestation, and diminished genetic diversity stand as the key concerns for dryland farmers [7]. When soil accumulates higher concentrations of salt that impede plant growth within the root zone in areas without irrigation, it leads to dryland salinity. The movement of salt from saline soil often triggers subsequent effects on water resources downstream, leading to the loss of accompanying infrastructure, environmental assets, and social values. Salinity can directly harm agricultural systems [8,9,10].

Bioavailability and bioaccessibility were suggested to distinguish between the quantity of a chemical compound that effectively crosses an organism’s cell membrane and its potential to enter the organism. Diverse researchers employ different approaches, such as the area under the curve (AUC) technique or concentration ratios in particular organ intakes, resulting in incomparable bioavailability outcomes [11]. Nevertheless, this technique faces many challenges, such as a lack of standardized protocols and the need for better a integration of multi-disciplinary techniques. Other techniques used include reconnaissance maps to identify sediment samplings, personal observation methods, and taking pictures of the site conditions [12]. These techniques, although useful for representing and identifying the soil type, cannot be used to indicate in situ what type of soil an area has. For this, a subsequent study must be carried out, which requires time and skilled personnel. Due to its properties, soil can be identified using colorimetry techniques. The color of soils and sediments has long been regarded as a fundamental factor and a primary visual attribute for characterizing, describing, and comparing these geological substances. The hues of sediments and soils primarily fall into two ranges: (a) green-gray to red and (b) olive-gray to black [13]. Despite that, it is necessary to have the presence of qualified personnel and laboratory-based analysis, which consumes a considerable amount of time.

Until now, the mapping and surveillance of soil have depended on four primary methods: expert judgment, biophysical modeling, the traditional field-based method, and satellite observation [14,15]. In recent years, the use of the Internet of Things (IoT) and wireless sensor networks (WSN) has become a real option for soil monitoring in the framework of smart agriculture [16]. These expert-based methods involve subjectivity, variability, and challenges in ensuring accountability [15,17]. The traditional on-site method involves practices like regional sampling and visual inspections like diagrams and volumetric assessments. The advantage of this method is its ability to offer comprehensive, factual data regarding soil deterioration on a plot-by-plot basis. Regardless, this method is frequently criticized for being time-consuming, labor-intensive, expensive, and only applicable to small areas. Through the integration of Synthetic Aperture Radars (SARs) and optical sensors [18], it becomes feasible to ascertain the surface characteristics of the soil under observation. Similarly, passive sensors providing information about ultraviolet, visible, and infrared wavelengths of the electromagnetic spectrum possess limited spatial resolution, offering data solely from the soil’s surface [19,20]. Regarding the WSN and IoT monitoring solutions, their applications are mainly limited to soil moisture [16].

As a matter of fact, sensors like red, green, and blue (RGB)-based sensors have the capability to discern various environmental parameters. Within agricultural crop fields, color or RGB images play a crucial role in identifying signs of diseases, shortages in fertilizer, damaged vegetation, and various weed and plant species. In RGB imagery, an object’s color results from the interplay between reflected light from the source and its optical traits, perceived by human vision. In farming, processing RGB-based images has proven effective for tasks such as weed identification, visualizing fields, the optimized use of fertilizer, the classification of soil texture, delineating physiological processes around plant surfaces, measuring plant height, and counting plant stands [21,22,23,24,25]. Nevertheless, while applicable to soil, these sensors cannot provide insights into the soil’s internal composition. Different applications of RGB sensors can be explored through the utilization of RGB Light-Emitting Diodes (LEDs). In employing this sensor, it becomes feasible to detect suspended particles in water by directing a light beam from a sensor to a Light-Dependent Resistor (LDR), gauging the water’s transparency [26]. Nevertheless, this approach is limited to aquatic settings and cannot be utilized elsewhere.

The aim of the paper is to develop a low-cost RGB-based sensor to be integrated into a WSN capable of detecting changes in the composition of the soil designed for dry agriculture. To achieve this goal, a combination of three different materials together with the soil has been carried out. The substances used to produce the mixtures were salt (sodium chloride: NaCl), sand, and nitro phosphate (NO_6_P^−2^) in the form of ammonium nitrate phosphate (H_16_N_5_O_7_P). By using a guide to redirect the beam of light, it has been possible to observe whether the light beam, when reflected on the wall of a grounded tank, is able to receive different values at the LDR photoreceptor. The data obtained by the LDR receptor will be processed and analyzed. Furthermore, this study will provide easier and faster methods for the identification of substances in soil. As a classification method, discriminant analysis (DA) is used to generate a series of centroids which will be included in the sensor node coding. Thus, the node will be able to classify new data without the need for running machine learning classification algorithms. These innovations will provide not only high-speed and precise identifications of substances in soil, but also remote and automatic techniques for soil management.

The rest of the paper is structured as follows: Section 2 outlines the related work. The proposed system is fully described in Section 3. Following this, Section 4 details the test bench. The results are presented in Section 5. Section 6 discusses the results obtained in comparison with other authors and possible solutions. Finally, Section 7 summarizes the conclusion and future work.

## 2. Related Work

This section provides an overview of antecedent research endeavors pertaining to the non-destructive classification of soil. Subsequently, based on the identified limitations of existing scientific studies, a potential motivation for investigating a low-cost RGB sensor as a potential soil classification system to address and overcome some of these challenges is proposed.

One of the most common methods of soil classification is image analysis. In this regard, a study aimed to achieve cost-effective and quick soil-type prediction was undertaken using a smartphone camera. The authors employed RGB extraction, V extraction from hue, saturation, and value (HSV) bins, and adaptive histograms to highlight the texture features. As a result, a novel lightweight convolutional neural network called Light-SoilNet, with an overall accuracy of 97.2% in classifying five soil types, sand, clay, loam, loamy sand, and sandy loam, was reported. Nonetheless, the proposed model might require a diverse and representative dataset for accurate predictions and could face challenges associated with the real-time implementation of the proposed model in agricultural settings [27]. Similarly, another study explored and compared the roles of visible-spectrum and machine learning vision for soil classification based on a smartphone-based soil-color classification sensor. Soil images were converted to RGB signals which were further processed, resulting in an accuracy of over 90% in the discriminant analysis. Although the proposed model addressed the portability and ease of access of the proposed system, challenges in varying light conditions might need to be addressed. Furthermore, the utilization of this model necessitates the drying of soil samples as a preliminary step, potentially posing challenges for real-time or rapid applications [28]. Another interesting study employed RGB image soil-color analysis and a feed-forward backpropagation neural network (ANN) to make decisions on whether to irrigate the soil [29]. They took images at different distances, time intervals, and levels of illumination throughout a four-week data acquisition period, exploring the water requirements for loam soils. The results indicated mean square error values of 1.616 × 10^6^ for training, 1.004 × 10^5^ for testing, and 1.809 × 10^5^ for validation. Nevertheless, the study might consider the need for validation across different soil types and conditions. Environmental factors could also be important to consider for the scaling-up or real-world deployment of this system. Whereas, a novel analytical method for predicting and classifying soil texture (i.e., clay and sand) using multivariate image analysis of 63 soil samples, grinded and sieved to <2 mm particle size, was reported. Authors achieved a 100% match in the classification and prediction of soil texture when digital image processing combined with multivariate image analysis was employed [24]. 

Considering the use of non-invasive sensor technologies for the classification and prediction of fertilizers or nutrients, various studies have employed statistical and machine learning approaches. For example, a study employing three types of sensors (JXBS-3001, FC-28, and DHT11) introduced a framework specifically devised for the prediction of three essential soil fertility components—organic matter (OM), potassium oxide (K_2_O), and phosphorus pentoxide (P_2_O_5_) based on 400 soil samples. This framework employed three distinct machine learning methodologies: multiple linear regression, support vector machine (SVM), and random forest (RF). The results demonstrated the exceptional performance of the CatBoost classifier in this context, achieving an accuracy rate of 97.5%, a precision value of 98%, a recall rate of 96%, and an F1-score of 97.5%. This underscores the classifier’s efficacy in providing accurate classifications and informs the development of a robust fertilizer recommendation model for improved soil fertility management strategies [30]. A study focused on the effects of varying urea–nitrogen fertilizer application rates on volatile organic compound (VOC) emissions investigated the use of an experimental metal oxide semiconductor (MOS) electronic nose that was based on eight sensors of the MQ and TGS series [31]. To be precise, the doses of urea fertilizer used on cucumber plants were 0, 100, 200, 300, and 400 kg/ha. The results showed that quadratic discriminant analysis (QDA) was the method of choice with an overall accuracy of 98.67%, among linear discriminant analysis (LDA), SVM, and ANN. Similarly, in another study, a classification accuracy of 95% and 97.78% was reported in discerning 12 distinct groups of basil plants through the employment of LDA and QDA methodologies, predicated on the application of urea fertilizer [32]. This investigation represented a unique nitrogen-related study pertaining to basil, wherein the classification is contingent upon the quantity of nitrogen fertilizer administered, facilitated by the utilization of an electronic nose. The study incorporated diverse analytical approaches, namely artificial neural networks, principal component analysis, linear resolution analysis, and quadratic statistical analysis, to comprehensively examine the acquired data. Whereas, a Two-Coil Systems (TCSs) sensor based on the electromagnetic principle was developed by [25], in which the proposed model was reported to successfully predict fertilizer concentration using an ANN model, achieving 100% correctly classified cases. Nevertheless, the existing studies on fertilizer classification for fertilizers or nutrients lack simplicity and fail to offer cost-effective solutions. The data acquisition procedures reliant on the employed sensors are susceptible to diverse environmental influences. Furthermore, the majority of these studies utilize intricate data analysis methodologies and machine learning approaches, applicable only within the confines of the proposed models.

Monitoring salinity dynamics is crucial for decision making for ensuring higher gains on crop production. In this regard, a study employed a digital camera for the estimation of soil salt content and soil surface roughness, thereby developing a four-color component (red, green, blue, and gray)-based prediction model [33]. The results showed an overall accuracy (R^2^) of 0.90 and 0.71; and a ratio of performance to deviation (RPD) of 3.11 and 1.87 for soil salt content and soil surface roughness, respectively. Furthermore, Jahangeer et al. [34] employed HYDRO 21 and TEROS 12 sensors for measuring conductivity to track surface water salinity and soil salinity in six selected saline wetlands. The results obtained through the Pearson correlation, utilized to assess the relationship of soil salinity with soil moisture (water content) and soil temperature, identified the degradation of topsoil by increasing salinity. Whereas, another study predicted soil electrical conductivity (EC) using various smartphone-based color coordinates (RGB, HSV, and CIE Lab*) and individual or combined visible–near infrared (Vis–NIR) and portable X-ray fluorescence (pXRF) spectra [35]. The results showed that combined Vis–NIR and pXRF spectra exhibited the highest prediction accuracy (R^2^ = 0.93) for predicting EC, surpassing individual Vis–NIR or pXRF spectra and smartphone-based and Vis-based color coordinates. Likewise, surface soil salinity in coastal areas using digital photographs was reported with an overall accuracy of R^2^ = 0.75, RMSE = 3.52, RPD = 2.02 based on the extraction of three-color components from RGB color space and five color spaces (HIS, CIEXYZ, CIELAB, CIELUV, and CIELCH) through color space conversion [36]. Specifically, authors conducted a correlation analysis between soil EC and color parameters to develop a soil EC estimation model using RF and leave-one-out cross-validation with 70% of the dataset for training and 30% for validation. In addition, color parameters extracted from digital images were found to be an effective approach for soil salinity estimation. Nonetheless, these studies on the monitoring of soil salinity encompass challenges in generalizability across diverse environmental contexts, potential issues related to the size and representativeness of training datasets, reliance on correlated parameters without establishing causation, sensitivity to temporal variability, calibration and maintenance requirements for sensors, concerns about model overfitting, resource intensity in terms of equipment and expertise, and the potential sensitivity of remote-sensing techniques to environmental factors. Additionally, complexities in model interpretation, data privacy, and security considerations in smartphone-based data collection present noteworthy challenges. A tabular summary of the most recent and relevant studies, providing an overview of the previous work, is presented in Table 1.

In light of the previously proposed systems as outlined above, the incorporation of an RGB sensor holds promise in mitigating concerns associated with environmental variables, scalability, and practical applicability in real-world scenarios. In contrast to certain specialized sensors that may encounter difficulties across diverse situations, the inherent versatility of an RGB sensor positions it as a prospective candidate for soil classification within varied agricultural settings. The exploration of a low-cost RGB sensor is motivated by the objective to furnish a more economically viable and easily accessible solution for soil classification, with potential validation across diverse environmental conditions and soil typologies. The RGB sensor, functioning by capturing data within visible light wavelengths, offers a blend of simplicity and affordability. This strategic utilization of an RGB sensor has the potential to surmount challenges linked to specific frequency ranges, thereby enhancing the practicality of the system for widespread deployment in real-world agricultural contexts.

## 3. Sensor Proposal

In this section, we elaborate on the proposed system designed for the analysis of sample composition. Initially, we provide a comprehensive sensor description, encompassing the details of all included devices. Subsequently, we identify the specific sensor and nodes utilized in the system. Finally, we present the architecture of the proposed system in detail. 

### 3.1. Sensor Description

The system consists of a union between an RGB light transmitter and an LDR light sensor, whose overall prices oscillates between EUR 0.99 and 2. The technical detail of each sensor is described. The transmitter is a digital sensor, with an operating voltage of 5 V. Its weight is approximately 1 g, with its dimensions being 2 cm long and 1.5 cm wide. The LDR light sensor is an analog sensor integrated with a 10 kΩ in-line resistor and an operating voltage between 3.3 and 5 V. Its weight is approximately 1 g, with its dimensions being 2 cm long and 1.7 cm wide. The disposition is as follows. The RGB transmitter and LDR sensor will be placed next to each other. Normally, these devices are placed opposite to each other, in order to measure the amount of light passing through a sample. Nonetheless, by having a solid, opaque piece in the center, the light beam might not be able to pass through it. That is in accordance with the law of reflection, which dictates that the angle of reflection is equal to the angle of incidence. 

**Figure 1:**
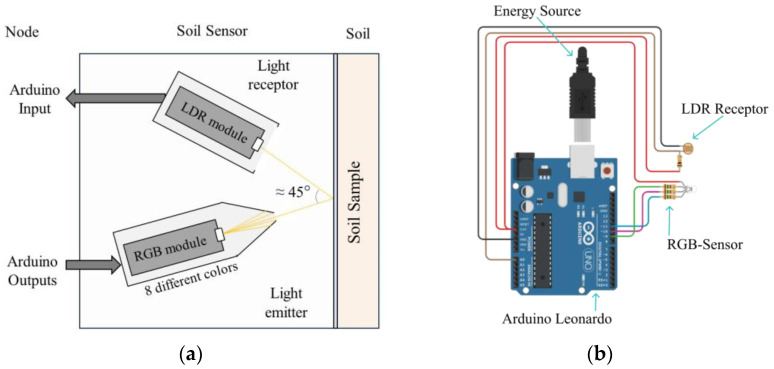
Schematic representations of the proposed sensor disposition. (**a**) Schematic representation of the proposed RGB-based sensor system where light emitted from RGB sensor is illustrated to be received by Light-Dependent Resistor (LDR). (**b**) Illustration of the proposed sensor node consisting of RGB sensor and Light-Dependent Resistor (LDR) photoreceptor.

Therefore, our proposed system places the RGB and LDR modules as shown in Figure 1a. This arrangement was selected since placing the devices at a 0° angle was not possible due to the sensors’ large sizes and to avoid equipment overheating. By doing so, the light beam will be emitted from the RGB, hit the cuvette wall, and bounce back to the LDR receiver. Both the RGB and the LDR receiver are positioned at an approximate angle of 45°, to smoothen the path for the light beam to reach its target.

### 3.2. Selected Node

An Arduino Leonardo node is selected for this application. This node is used because of its slightly high computational capacity compared with Arduino ONE. There is not much difference between these two Arduinos; nonetheless, we opted for Arduino Leonardo. This node was selected, in comparison to Arduino ONE, first of all, because of its slightly lower price, around EUR 21, the increase in pin numbers, its 32 KB flash memory, which has 4 KB used for the bootloader, and its improved communication capabilities. On another note, Arduino Leonardo has an ATmega32u4 microcontroller (Microchip Technology Inc., Chandler, AZ, USA), with an operating voltage between 7 and 12 V, a weight of 20 g, a 7 cm length and a 5.5 cm width.

The system is based on a series of sensors and receptors connected to the node, as can be seen in Figure 1b. The suggested method for powering the system involves using a USB–A connection, as previously illustrated in Figure 1b. Additionally, external batteries and other relevant energy sources can be employed for this purpose. The RGB light emitter is followed by the LDR light photoreceptor, both connected to the Arduino Leonardo node; see Figure 1b. Subsequently, the data obtained were stored via the node and were used to classify the data using edge computing. Finally, the data are forwarded through the network. The communication technology can be adapted to the requirements of the network in terms of distance between nodes and the required bandwidth for other additional purposes.

### 3.3. Architecture

**Figure 2:**
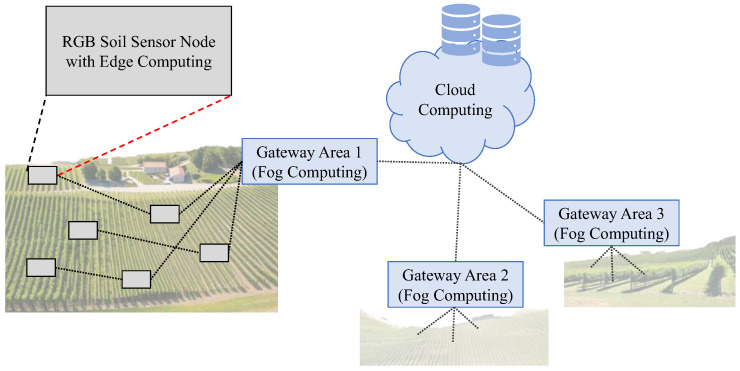
The architecture of the sensing system with RGB-based soil sensors.

The proposed sensor will be part of a broader proposal for an agriculture monitoring system based on IoT and WSN. The sensor node, which is endowed with an edge computing capacity, will forward the data to a smart gateway endowed with a Fog Computing capacity. While on the edge, the data will be processed to calculate the soil composition locally, reducing the amount of exchanged information. In the gateway, data fusion and data comparison will be performed with the rest of the nodes in the network, which corresponds to a given portion of the territory. Thus, the gateways will smartly reduce the energy use by fusing data and will provide a fast response detecting abnormal situations based on the forwarded data from the sensor nodes. Finally, in cloud computing, artificial intelligence and machine learning will be applied to provide predictive responses. The architecture can be seen in Figure 2. 

## 4. Test Bench

This section provides a comprehensive account of the tasks carried out to assess the effectiveness of the proposed sensor model. It begins with a detailed description of the soil preparation, followed by the disclosure of the prepared sample mixtures and their respective proportions. Lastly, the process for acquiring and analyzing data is outlined.

### 4.1. Soil

The sampled soil was sourced from a field near the Cordoba city of Spain (37°54′07.8″ N 4°48′31.8″ W). The collected soil exhibited an Alfisol composition as per the classification of the National Geographic Institute (IGN) of Spain [37]. The soil samples were ground to the finest consistency achievable employing a mortar and pestle. The unwanted material including rocks, plant stems, leaf molds, soil lumps, etc. were removed manually from the soil. The resulting soil was sieved through a 2 mm fine mesh strainer. This filtration process was carried out to obtain a homogenous particle size of the soil. The samples were then weighed on a weighing balance and poured into 1 mL plastic cuvettes with dimensions of 12.5 × 12.5 mm. 

### 4.2. Added Substances

Three distinct materials—sand, NaCl, and nitro phosphate—were blended with soil in a specified ratio as represented in Table 2. A control group was established by treating soil devoid of additional substances. Meanwhile, varying proportions of sand, NaCl, and nitro phosphate were amalgamated, yielding four distinct concentrations (Table 2). To ensure consistency, a constant weight of the soil was maintained for NaCl and NO_6_P^−2^ mixtures owing to their powdered form. In the case of sand samples, characterized by granular-sized particles, an adjusted weight proportionate to the concentration was employed. Each sample had three replicates for each concentration. Particle sizes equivalent to those of sieved soil particles or similar dimensions were utilized for each material. The samples underwent thorough mixing in a mechanical manner using a mortar and pestle, after which they were weighed on a laboratory-scale weighing balance. Subsequently, the meticulously homogenized mixtures were transferred into plastic cuvettes for data reading and collection.

### 4.3. Data Gathering Process 

**Figure 3:**
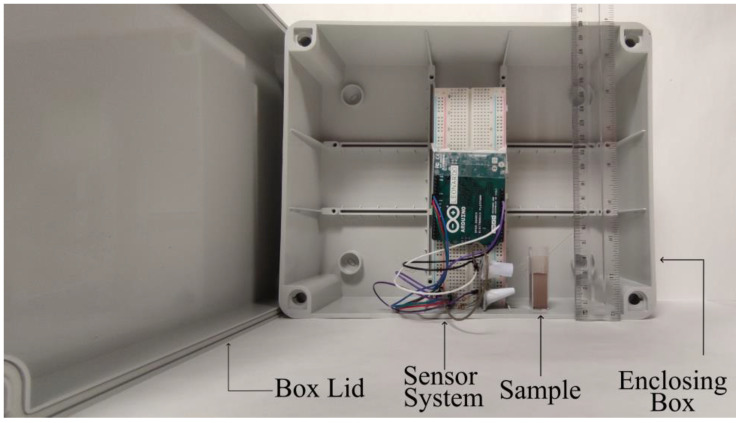
Representation of the proposed sensor system.

The prepared samples, previously poured into the glass cuvette, were individually positioned in close proximity to the sensor (Figure 3). To optimize the reception of reflected light, the RGB sensor and the receiving unit were strategically arranged at an approximate angle of 45°, taking into account that the maximum signal reception was observed by the employed photo-receptor module. A light-tight system was established to eliminate any interference from external light sources, accomplished by enclosing the sample within the sensor-mounted box. The Arduino Leonardo is employed to program the various colors of the RGB LED. To prevent interference from the LEDs and infrared (IR) illumination on the samples, data collection is avoided during the primary instances of the execution. The collected data are stored in a CSV format and subsequently undergo processing. Data recording involved three repetitions for each sample, with three replicates employed for each instance.

### 4.4. Data Processing 

The data obtained from the sensor was documented in an Excel spreadsheet. Subsequently, data normalization was carried out, referencing the maximum and minimum achievable light absorption by the employed RGB sensor. Following normalization, average values were computed for all repetitions. This was succeeded by an analysis of variance (ANOVA) and a subsequent discriminant analysis (DA). The Statgraphics 19 program was used for the statistical analysis. 

## 5. Results

Within this section, we elucidate the acquired findings. Initially, we present the outcomes stemming from a comprehensive analysis utilizing RGB sensor lights on the sample material. Subsequently, we document the results derived from the classification and verification processes applied to the samples.

### 5.1. General Analyses

The collected data were transformed initially. It is crucial to emphasize that from an analytical standpoint in edaphology, the process of data normalization was undertaken. Specifically, the initial values obtained from the LDR receptor were mathematically transformed to conform to a scale ranging from one to three, as represented in Table 3. This data normalization was applied to all the recorded data. As a consequence, it serves to present the signal or data in a manner analogous to that perceived by a spectrophotometer.

**Figure 4:**
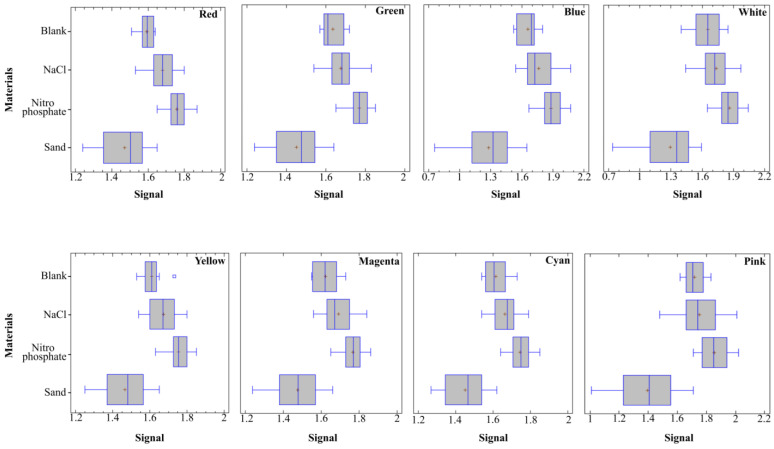
Summary of average values perceived by Light-Dependent Resistor (LDR) receptor for each material for eight colors (red, green, blue, white, yellow, magenta, cyan and pink) of RGB sensor.

Afterwards, a comprehensive analysis, wherein the calculation of the average values was executed by considering the individual color readings from the RGB sensor. This calculation took into account both the nature of the material under investigation and the percentage concentration of each sample material (Figure 4). The pink color, out of all these colors, was not capable of correctly identifying the samples, whereas the rest of the colors were found to identify the samples based on their corresponding concentrations. Nevertheless, this could be further evaluated by undertaking an ANOVA.

Subsequently, an ANOVA was systematically performed across all tested materials, i.e., salt, sand, and nitro phosphate. The resultant findings were meticulously categorized based on the discriminative capacity of each individual color to accurately classify samples according to their respective concentrations. Furthermore, emphasis was placed on evaluating the significance of the obtained results through the consideration of *p*-values.

In the specific case of salt samples, an intricate examination revealed magenta to exhibit the most prominent and statistically significant values in sorting samples by concentration followed by red, blue, yellow, and white colors (Table 4). Nonetheless, the results derived from the classification based on green, cyan, and pink light were deemed statistically insignificant.

Considering the sand samples, the ANOVA results identified the green and yellow colors as yielding statistically significant sets based on sample concentrations (Table 5). Additionally, cyan and pink emerged as the third and fourth most outstanding colors, respectively, in accurately classifying samples according to their concentration levels. Whereas, red, blue, white, and magenta did not yield any significant values.

When nitro phosphate samples were evaluated, it was discerned that magenta stood out as the sole color demonstrating promising results in correctly grouping samples based on their respective concentrations (Table 6). Despite this, the inclusion of yellow and green colors in the analysis was deemed pertinent, not only owing to their classification efficacy in salt and sand samples but also to further assess their broader applicability in the analytical framework. Whereas, the rest of the colors did not significantly classify the samples.

### 5.2. Classification of Subtances

In order to test whether the observed values obtained above are significant, bar charts were generated together with the materials. This was intended to determine the minimum number of colors needed to evaluate the soil composition, thus saving energy in the developed system.

**Figure 5:**
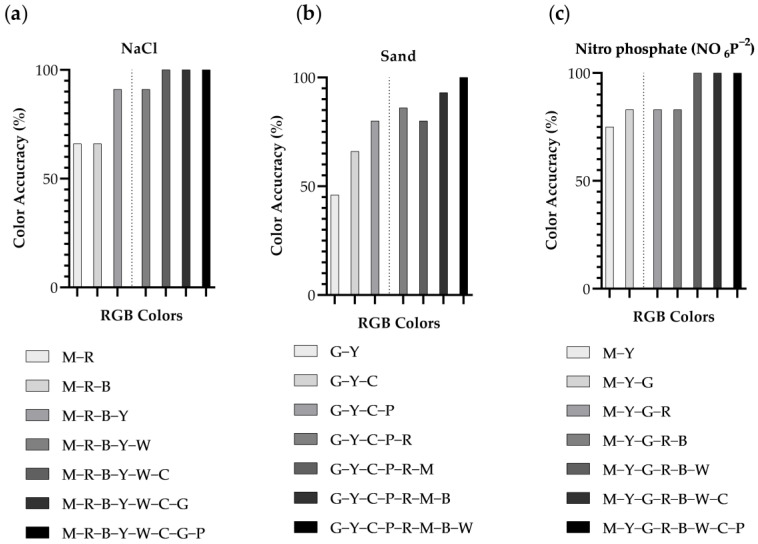
Summary of classification accuracy percentage (%) for NaCl (**a**), sand (**b**), and (**c**) nitro phosphate (NO_6_P^−2^) based on RGB-sensor characteristics. The dotted line marks the separation between the selected and discarded colors, where: R: red; G: green; B: blue; W: white; Y: yellow; M: magenta; C: cyan; P: pink.

In the case of NaCl, as can be seen in Figure 5a, using the first two or three colors, as explained in the ANOVA classification, we obtained a hit rate of 66.67%. Additionally, if we were to select the first four and five colors, we can see that it would rise to a 91.67% classification accuracy percentage. And finally, by adding the rest of the colors, we obtained a hit rate of 100%. In this case, we should use the colors magenta, red, blue, and yellow, since they reach the same hit value as if we were to add white.

In the case of sand, if we look at Figure 5b, if we use the first two or three colors, as explained in the ANOVA classification, we obtain a classification accuracy percentage of 46.67% and 66.67%, respectively. On the other hand, if we add one more color, in this case, pink, we ascend to 80% of correctly classified cases. Nonetheless, if we were to add the red color, the value would increase to 86.67%. It is true that, if the colors magenta and blue were added, we would obtain a success rate of 93.33% of classified cases, although the energy required to operate the system would be very high. And finally, if all the colors were added, we would obtain a classified case accuracy of 100%. In this case, we would select the simplest color option: green, yellow, cyan and pink. 

In the nitro phosphate case, if we look at Figure 5c, in putting together the selected colors magenta and yellow, we would obtain a classification accuracy percentage of 75%. Additionally, if we were to add the colors green, red and blue, we would obtain a success rate of 83.33% of classified cases. Finally, if we were to add the rest of the colors, the accuracy would increase to 100%. In this case, since adding only magenta, yellow, and green yields the same result as adding more colors, it was decided to keep this option.

**Figure 6:**
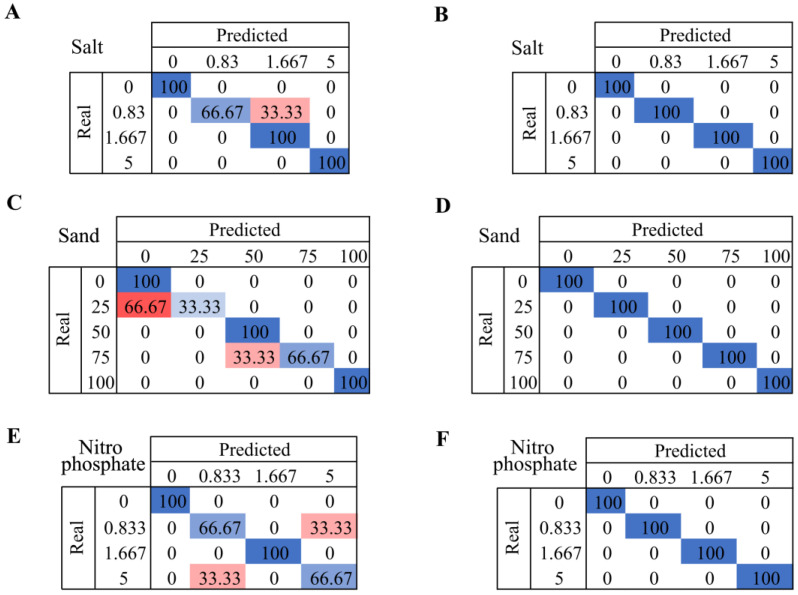
Summary of confusion matrices, highlighting accurately predicted cases with least errors using the minimum color combinations compared to the ideal accuracy of 100%. For salt, the least-error cases were based on magenta, red, blue, and yellow (**A**), and those of 100% accuracy were for magenta, red, blue, yellow, white, and cyan (**B**). For sand, the least-error cases were based on green, yellow, cyan, and pink (**C**), and those of 100% accuracy were for green, yellow, cyan, pink, red, magenta, blue, and white (**D**). For nitro phosphate, the least-error cases were based on magenta, yellow, and green (**E**), and those of 100% accuracy were for magenta, yellow, green, red, blue, and white (**F**).

The classification results with DA can be seen in Figure 5. Confusion matrixes are used to compare the results of the previous analysis. It contains the confusion matrixes using the thresholds obtained with the ANOVA for salt, Figure 6A,B, sand, Figure 6C,D, and nitro phosphate, Figure 6E,F.

To identify the presence of salt, the colors chosen on the DA to obtain this confusion matrix were magenta, red, blue, and yellow. In this case, it is possible to detect the presence of this material at concentrations of 0, 1.667, and 5 with 100% accuracy. On another note, at a concentration of 0.83, the system generates a lesser confusion with a concentration of 1.667, as can be seen in Figure 6A,B, which represents the confusion matrix when the fewest number of colors are added. In this case, we added the same number of colors as above, along with two significant colors, although, to reach 100%, one last non-significant color was added, cyan. This color does not meet the classification rules described in Section 5.1, but it has been observed that by adding it, 100% of the correctly classified cases are reached.

Whereas, Figure 6C corresponds to the confusion matrix of sand. In this case, the colors used were green, yellow, cyan, and pink. It is possible to identify cases with a 0%, 50%, and 100% presence of sand. Nevertheless, it presents difficulties in determining whether the sample has a 0% or a 25% concentration. Furthermore, the system presents a lesser error in identifying whether the sample has a 50% or 75% concentration of sand. On the other hand, Figure 6D represents the confusion matrix for all colors in order to obtain a 100% success rate for classified cases. In this scenario, it was necessary to add four non-significant variables to the matrix.

To conclude, the nitro phosphate confusion matrix is represented in Figure 6E. The colors used in this DA were magenta, yellow, and green. With the combination of these colors, it was possible to identify all cases with a concentration of 0 and 1.667 of nitro phosphate. Therefore, the system tends to have lesser confusion with concentrations between 0.833 and 5. Furthermore, Figure 6F represents the confusion matrix when the fewest number of colors are added. In this case, the combination of magenta, yellow, and green was necessary. Nonetheless, to achieve a 100% success rate for classified cases, three non-significant colors were implemented: red, blue and white.

## 6. Discussion

In this section, we detail the main implications of the proposed system and the obtained results. First of all, a comparison with existing similar systems is presented. Then, the main impact and possible applications of the proposed system are depicted. Finally, the detected limitations of the presented data are evaluated and justified.

### 6.1. General Findings

A discussion of the obtained results with existing solutions is shown in this subsection. Accuracy is the main metric used to compare the performances of the monitoring systems. The subsection is divided into three subsections. In the first two, the results for soil texture and the presence of different substances in the soil (salt and fertilizer) are contextualized within the current monitoring framework. Then, in the third subsection, a summary of the advantages of the proposed solution is highlighted. 

#### 6.1.1. Soil Texture Recognition

Concerning the use of sensors for identifying soil texture, the following examples offered results that can be compared with the proposed system. Among the different approaches followed, the one with a higher accuracy is the use of images based on the use of artificial vision [24,27]. Even though these methods attain high accuracy percentages, from 97.2% to 100%, their high computation requirements of data that must be computed in the edge make them less efficient than the proposed system. The alternative of sending the image to the cloud to compute it beyond the network will require a network with an adequate bandwidth and the use of energy to send images through the network. Moreover, the use of cameras to gather images in underground sensor networks will require different lighting elements. Considering that when the proposed system uses all the lights, achieved accuracy reaches 100%, the use of the proposed method, with less computational and network requirements, presents an advantage.

Other authors presented proposals similar to the approach used in this paper based on light spectra analyses. In [38], the authors used visible (Vis) and near-infrared (NIR) data, while in [39], they also used pXRF information. In both cases, accuracies were below 100%, ranging from 70% to 90% with Vis and NIR data and 97% when X pXRF-ray information was added. Additional examples can be found identifying the % of sand and clay contents using VIS and NIR data in [40,41], or including pXRF data [42], but no information on accuracies is presented. The used metrics include R^2^, all of them below 0.88 [40], 0.90 [42], and 0.95 [41]. Thus, we can affirm that the proposed method poses advantages beyond the state of the art in soil texture identification; see Table 7 for a summary of the aforementioned information. 

Considering the different studied samples, in all cases, the authors used natural samples [24,27,28,39,40,42]. In this paper, artificial samples were generated to ensure a wide variety of cases to evaluate the sensor’s performance in different scenarios, having a variation from 0 to 100% clay and sand. Moreover, since the same soil was used, we reduced the noise and side effects due to organic matter and other possible interferents. This also allowed us to generate replicas, which provide more solid results. The number of classes ranges from four [37], five [27], and six [24]. In our case, five classes were used. In most cases, the used classes correspond to the texture of the soil. In our case, the class corresponded to the percentage of sand and clay in the sample. In [38,40,41,42], the authors also used the percentage of soil components as a predictor.

Among different data-processing techniques in the included studies, there is a wide variety of methods, such as convolutional neural network (CNN) [27], RF [39], partial least squares regression (PLSR) [38,40,41], and multivariate image analysis (MIA) [24]. In some cases, even two different methods are combined, such as recursive feature elimination (RFE) with RF [42]. None of the included ones used DA. In [28], DA was used to predict the soil type but not the soil texture.

#### 6.1.2. Salinity and Fertilizer Quantification

As in the previous case, there are different methodologies for evaluating salt content in soil. First of all, we compare our results with those proposals based on image analysis. In [9,43], different methods based on image analysis were proposed and evaluated. The proposals based on image analyses are characterized by good performance by an R^2^ of 0.9 [9] and 0.73 [42]. As in the previous case, spectroscopic methods are also used to determine soil salt content. In [44,45], the conjunction of NIR and Vis spectra were used as inputs for evaluating the electroconductivity (EC) of soil and soil saline content (SCC), and the obtained R^2^ reached 0.99 [44] and 0.87 [44,45] for the best combination. In some cases, image processing is also combined with spectroscopic information [35]. In this case, accuracies given as R^2^ are 0.7 for data from the smartphone and 0.93 for pXFR and Vis–NIR data; see Table 8. All these cases offered a performance of their results in terms of R^2,^, which makes the comparison impossible. Nevertheless, in our case, using six lights achieved an accuracy that reached 100%, making this a promising solution.

Concerning the detection of the soil nutrients of fertilizers, existing solutions are based on remote sensing and evaluating the plant’s status. Few options focus on determining the content of the fertilizer or a given nutrient based on soil monitoring. Among those papers, one is based on multiple soil sensors [30] and two are based on spectroscopic data, including NIR–Vis [46] or a low-cost color sensor [47]. Achieved accuracies ranged from 80 [46] to 100 [47] using spectroscopic data and 97% using soil sensors [30]; see Table 8. The proposed system is similar to the one in [47], but this solution improves the accuracy levels, with results similar to complex methods based on NIR–Vis data. 

Considering the different studied samples, in all cases, the authors used natural samples [30,35,43,44,45,46]. Only in [47] were the analyzed samples artificially generated, as in our case. In most of the papers, the authors do not generate replicas. The only case in which replicas were generated is in [45], where five replicas were used. The number of classes for the classification algorithms ranged from three [47] to five [35,46]. There is a wide variety of classification and regression methods such as CNN [35], ANN [46], RF [35,43,44,46], support vector machine (SVM) [35,46], Gated Recurrent Units (GRU) [35], and Native Bayesian (NB). None of the included ones used DA. Among the most used regression tools, PLSR [9,43,44] is the most used, while others such as random forest regression (RFR), support vector regression (SVR), Gradient-Boosted Regression Tree (GBRT), Multilayer Perceptron Regression (MLPR), and Least Angle Regression (Lars) are used only in [45].

**Table 8:** Summary of solutions for SSC and fertilizer presence in soil.

Year	Data Type	Processing Technique	Parameter	Levels Min/ Max	No. of Classes	No. of Samples	Replicas	Type of Sample	Acc.	Ref.
2019	Images	PLSR	SSC (%)	0–0.5	-	49	-	Natural	-	[9]
2020	Images	PLSR or RF	SSC (%)	0.08–5.4	-	93	-	Natural	-	[43]
2023	Image pXRF, VIS, and NIR	CNN + RF+ SVM + GRU	SSC (%)		5	240	-	Natural	-	[35]
2022	NIR–Vis	PLSR, RF	EC (mS/cm)	0–10	-	231	-	Natural	-	[44]
2019	NIR–Vis	Multiple	SSC (%)	0.29–29.1	-	60	5	Natural	-	[45]
2024	VIS data of LDR	DA	SSC (%)	0.83–5	5	15	3	Artificial	100%	Proposal
2023	Soil sensors	CatBoost	Nutrients	-	-	-	-	Dataset	97%	[30]
2018	Color sensors	NB	Fertility	-	3	10	-	Artificial	80%	[47]
2021	UV–Vis	RF, SVM, ANN, NB	Nutrients (%)	0–4	5	58	-	Natural	100%	[46]
2024	VIS data of LDR	DA	Nitro phosphate (%)	0.83–5	5	15	3	Artificial	100%	Proposal

#### 6.1.3. Main Advantages of the Proposed Sensor

Among surveyed examples of sensing elements for soil sensor characteristics, the proposed ones offered a series of advantages. These advantages can be summarized as efficiency, real-time and in situ sensing, and the option of measuring different parameters with a single device.

**Figure 7:**
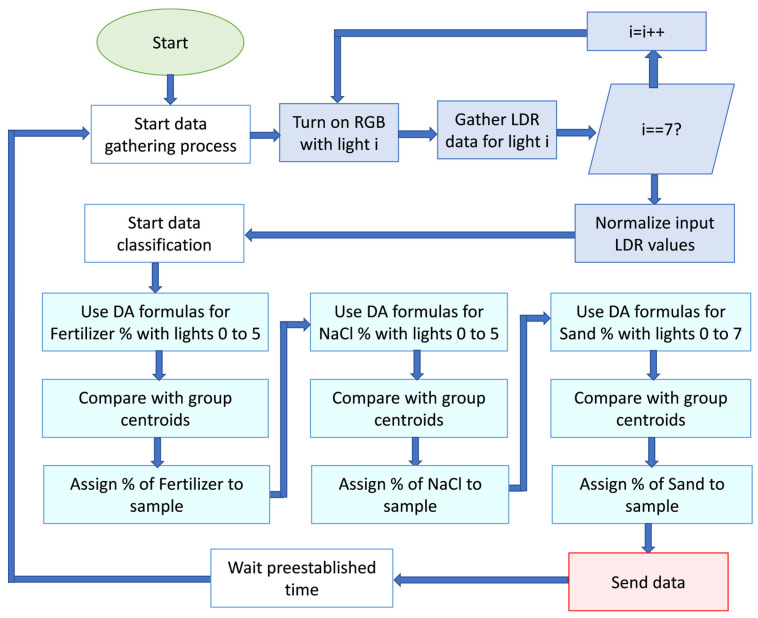
Operation of sensing process of the proposed sensor and data classification.

The most important one is the balance between accuracy and computational requirements. The achieved accuracies reached 100% for analyzed parameters by measuring light abortion for six different colors. Even though DA is used in this stage for the sensor operation, no machine learning capabilities are necessary. With the current results of the DA analyses, it is possible to include the DA functions in the node to calculate the DA coordinates for a given sample. Then, it is possible to compare it with the centroids of the pre-established groups and evaluate which is the closest centroid to assign the group to the new sample, as shown in Figure 7. Thus, the computational requirements are limited to applying two mathematical functions with six float variables to calculate the coordinates of the sample given the DA functions and calculate the distance between the new sample and the five given centroids.

Next, the proposed sensor is able to measure in real-time with an average measuring time of 6 s per sample. With the capability of being powered with an Arduino and using the microprocessor to process data, it is possible to use this sensor as an autonomous device in a wireless sensor network. Thus, the sensor can be placed underground, measuring the soil characteristics automatically and at different depths. The simplified node operation for data gathering and data analyses for soil characterization is shown in Figure 7. The lights are codified in the Arduino node from zero to seven following the following order: red, green, blue, white, yellow, magenta, cyan, and pink. 

Finally, the possibility of measuring different important parameters linked to soil fertility in a single device using only one sensor is a great advantage compared to existing sensors. The most similar case in which a sensor network is used for soil monitoring is [30,47]. Nevertheless, in [30], multiple sensors are used to measure the soil nutrients. In [46,47], only one sensor, similar to the one used in this paper, is used to estimate the quantity of different nutrients. Thus, our sensor is similar to some of the existing solutions but offers an improved accuracy. 

Even though it tries to reduce the data used for classification, which impacts the energy consumption, for the correct monitoring of sand, all the lights must be used. In previous papers, such as in [48], it has been possible to reduce the data dimension and save energy using a limited number of input parameters while the accuracy is maintained at 100%. In this case, this was only possible for NaCl and the fertilizer. Using the ANOVA results to identify the most suitable colors offered an accuracy of 85% in the case of NaCl and the fertilizer and 80% for sand. 

### 6.2. Limitations of Presented Results and Possible Future Solutions

The main limitation of the present study is linked to the reduced number of samples for each parameter compared with the state of the art. Nevertheless, a total of 39 samples were created and measured, with the samples corresponding to 13 different combinations of the three studied parameters (sand, NaCl, and fertilizer) with a given bare soil. An additional limitation is the fact that machine learning classification is used instead of linear regression, as is done by multiple authors. In this first stage of the sensor development, the classification in groups with different percentages of evaluated parameters was preferred, and the regression models with more samples will be calculated in future work. 

Regarding the efficacy of different light sources in analyte detection, it is imperative to recognize the complexity of analytical processes, which hinge on multiple factors and elude a one-size-fits-all solution. The performance of light sources is intricately linked to the color and size of particles within a sample, necessitating a nuanced understanding of their interplay. This paper’s central focus lies in determining the most effective combination of lights for enhanced analyte detection, acknowledging that a singular light source may not suffice for comprehensive analysis. Beyond the pursuit of optimal detection, our broader objective encompasses the reduction of energy consumption in sensor technologies. By streamlining and minimizing the number of lights employed, our research aims to contribute not only to the advancement of analytical methods but also to the sustainable evolution of sensor technologies.

Finally, the use of artificial samples compared to natural samples is an additional issue of the conducted tests. While most of the related work used natural samples, we have generated artificial samples in this paper. This procedure is only followed by the authors of [47]. Although it might seem a limitation, having artificial samples provides solid data, with the predefined concentration of the analyte (salt, fertilizer of texture) and low noise by unknown substances. Thus, we can ensure that the response of the sensors is directly related to the different concentrations of the added substances. Moreover, we ensured that samples were homogenized. In proposals with natural samples, aspects such as the presence of organic matter, soil moisture, or non-homogenized texture might provide inaccurate results. Therefore, we have chose to use artificial samples for the initial calibration and proof-of-concept for this low-cost technology, as done in [47], with the evaluation of the suitability of color sensors for determining soil fertility. In future work, after generating a complex database, it is recommended that researchers test the proposed system with natural samples. 

## 7. Conclusions

This study was conducted to develop a low-cost sensor system capable of detecting changes in the composition of the soil. Currently, the approaches implied for the study of soil composition and profiling have several limitations, such as standardized protocols for taking measurements, an integration of multidisciplinary techniques, an absence of in situ soil studies, and/or a lack of refined techniques. Therefore, studies implying rapid and non-invasive methodologies are needed. In this regard, the use of an optical sensor could be an important technique. Therefore, this study was undertaken to devise a non-invasive method for identifying and characterizing the soil with an RGB-based sensor system. 

The investigation involved the assessment of three distinct materials—sand, salt, and nitro phosphate—incorporated into soil at specified proportions utilizing the RGB sensor system proposed in the study. The statistical analysis substantiated the sensor’s effectiveness in segregating each material type into distinct groups with statistically significant outcomes. Notably, the proposed system, employing combinations of more than two colors, entails higher energy consumption but achieves superior accuracy in data interpretation. 

Thus, the proposed low-cost RGB sensor system provides an economically viable and easily accessible solution for soil classification, with potential validation across diverse environmental conditions. The RGB sensor, functioning by capturing data within visible light wavelengths, offers a blend of simplicity and affordability. This strategic utilization of an RGB sensor has the potential to surmount challenges linked to specific frequency ranges, thereby enhancing the practicality of the system for widespread deployment in real-world agricultural contexts.

Future work might involve the evaluation of not only the solid materials, but also the liquid substances, and the inclusion of gas sensors [48], to characterize the soil. Considering the need for more rapid and economical approaches for the determination of humidity, fertilizer, chemical components, and the presence of organic matter, an optimized setting for RGB sensors could be investigated. Likewise, given the specific characteristics of each material, adjustments of the angle of reflection and refraction might need to be determined. Similarly, studies involving the validation across diverse environmental conditions and soil typologies could be undertaken. Despite the use of these sensors and the selected arrangement, we have verified that we are able to classify the different types of components in soil, and it would be interesting to study how different types of arrangements might affect the classification of this type of sensor.

## Figures and Tables

**Table 1:** Summary of previous studies.

Study	Year	Main Findings	Methodology	Limitations/Challenges
[28]	2016	Over 90% accuracy in DA for soil classification based on smartphone images.	Conversion of soil images to RGB signal, DA.	Challenges in varying light conditions and the drying of soil samples as a preliminary step.
[24]	2019	100% match in soil texture prediction using multivariate image analysis.	Multivariate image analysis of soil samples.	Limited validation datasets, applicability to soils with higher silt contents not tested.
[29]	2021	Successful decision making about soil irrigation using RGB image analysis using ANN.	RGB image analysis, feed-forward backpropagation neural network.	Need for validation across different soil types and conditions, consideration of environmental factors for real-world deployment.
[27]	2024	97.2% accuracy in classifying sand, clay, loam, loamy sand, and sandy loam using a camera.	RGB extraction, V extraction from HSV bins, adaptive histogram, Light-SoilNet CNN.	Requires a diverse dataset, challenges in real-time implementation in agriculture.
[31]	2021	98.67% accuracy in VOC emissions analysis based on urea–nitrogen fertilizer rates.	MOS electronic nose, quadratic DA.	Data acquisition susceptible to environmental influences.
[32]	2021	95–97.78% accuracy in classifying basil plants based on urea fertilizer.	LDA, QDA, various analytical approaches.	Only detects the nitrogen volatile compounds in the plant.
[25]	2023	100% accuracy in predicting fertilizer concentration using an ANN model.	Two-Coil Systems sensor, ANN.	Lack of simplicity, susceptibility to diverse environmental influences.
[30]	2023	97.5% accuracy in predicting organic matter, potassium oxide, and phosphorus pentoxide in soil using CatBoost.	Multiple linear regression, SVM, RF, CatBoost classifier.	Constraints associated with data accessibility, susceptibility to errors, system intricacy, cost considerations, and maintenance.
[33]	2019	R^2^ of 0.90 for soil salt content and 0.71 for soil surface roughness prediction.	Digital camera, four-color component prediction model.	Limited generalizability to different environments, dependency on environmental conditions, and challenges in scaling up.
[36]	2021	Overall accuracy of R^2^ = 0.75 based on digital photographs for soil salinity	Digital photographs, correlation analysis, RF.	Generalizability challenges, concerns about model interpretation, data privacy, and security.
[35]	2023	R^2^ of 0.93 for predicting EC using Combined Vis–NIR and pXRF spectra.	Smartphone-based color coordinates, Vis–NIR, pXRF spectra, RF.	Challenges in generalizability, reliance on correlated parameters, sensitivity to temporal variability.
[34]	2024	Identified topsoil degradation by increasing salinity.	HYDRO 21 and TEROS 12 sensors, Pearson correlation.	Point-level salinity measurement and limited duration.

Where DA: discriminant analysis; RGB: red, green, blue; ANN: Artificial Neural Network; HSV: hue, saturation and value; CNN: convolutional neural network; VOC: volatile organic compound; MOS: metal oxide semiconductor; LDA: linear discriminant analysis; QDA: quadratic discriminant analysis; SVM: support vector machine; RF: random forest; VIS–NIR: visible–near infrared; pXRF: portable X-ray fluorescence.

**Table 2:** A summary of sample types with the corresponding concentrations of prepared sample mixtures.

Sample ID	Test	Concentration (%)/(Numbers)	Added Substance (g)			
			Soil	NaCl	Sand	Nitro Phosphate (NO_6_P^−2^)
1	Blank	0	3	-	-	-
2	Salt	0.83	3	0.025	-	-
3		1.67	3	0.05	-	-
4		3.33	3	0.1	-	-
5		5	3	0.15	-	-
6	Sand	25	2.25	-	0.75	-
7		50	1.5	-	1.5	-
8		75	0.75	-	2.25	-
9		100	0	-	3	-
10	Nitro phosphate	0.83	3	-	-	0.025
11		1.67	3	-	-	0.05
12		3.33	3	-	-	0.1
13		5	3	-	-	0.15

**Table 3:** Representation of data transformation.

Light	Blank		NaCl		Sand		Nitro Phosphate (NO_6_P^−2^)	
	Original	Modified	Original	Modified	Original	Modified	Original	Modified
Red	497.67	1.59	504	1.69	488.67	1.52	511	1.77
Green	510.33	1.61	504.67	1.64	494.67	1.51	516.33	1.78
Blue	555.33	1.73	556.33	1.72	541	1.40	567.67	1.96

**Table 4:** Summary of analysis of variance (ANOVA) for salt samples.

Light	Percentage of Concentration					*p*-Value
	0	0.83	1.67	3.33	5	
Red	1.59 ^a^	1.66 ^ab^	1.69 ^b^	1.67 ^b^	1.71 ^b^	0.013
Green	1.64 ^a^	1.66 ^a^	1.63 ^a^	1.67 ^a^	1.74 ^b^	0.011
Blue	1.66 ^a^	1.70 ^a^	1.72 ^a^	1.75 ^ab^	1.87 ^b^	0.014
White	1.65 ^a^	1.69 ^ab^	1.71 ^ab^	1.72 ^ab^	1.82 ^b^	0.147
Yellow	1.61 ^a^	1.66 ^ab^	1.64 ^ab^	1.68 ^b^	1.71 ^b^	0.052
Magenta	1.62 ^a^	1.66 ^ab^	1.66 ^ab^	1.69 ^bc^	1.75 ^c^	0.008
Cyan	1.62 ^a^	1.65 ^a^	1.62 ^a^	1.66 ^a^	1.72 ^b^	0.005
Pink	1.72 ^a^	1.71 ^a^	1.73 ^ab^	1.72 ^a^	1.84 ^b^	0.147

Where different letters indicate significantly different values.

**Table 5:** Summary of analysis of variance (ANOVA) for sand samples.

Light	Percentage of Concentration					*p*-Value
	0	25	50	75	100	
Red	1.59 ^a^	1.47 ^bc^	1.49 ^b^	1.54 ^ab^	1.38 ^c^	0.001
Green	1.64 ^a^	1.47 ^b^	1.46 ^b^	1.51 ^b^	1.37 ^c^	0.0001
Blue	1.66 ^a^	1.28 ^bc^	1.27 ^bc^	1.39 ^b^	1.17 ^c^	0.0003
White	1.65 ^a^	1.27 ^bc^	1.34 ^bc^	1.40 ^b^	1.16 ^c^	0.0003
Yellow	1.61 ^a^	1.48 ^b^	1.51 ^b^	1.52 ^b^	1.34 ^c^	0.0001
Magenta	1.62 ^a^	1.49 ^b^	1.50 ^b^	1.53 ^ab^	1.38 ^c^	0.0002
Cyan	1.62 ^a^	1.48 ^b^	1.46 ^b^	1.50 ^b^	1.36 ^c^	0.0001
Pink	1.72 ^a^	1.42 ^b^	1.43 ^b^	1.43 ^b^	1.30 ^b^	0.0003

Where different letters indicate significantly different values.

**Table 6:** Summary of analysis of variance (ANOVA) for nitro phosphate samples.

Light	Percentage of Concentration					*p*-Value
	0	0.83	1.67	3.33	5	
Red	1.59 ^a^	1.77 ^c^	1.71 ^b^	1.76 ^c^	1.80 ^c^	0.0001
Green	1.64 ^a^	1.78 ^c^	1.72 ^b^	1.77 ^c^	1.80 ^c^	0.0001
Blue	1.66 ^a^	1.90 ^c^	1.89 ^b^	1.92 ^c^	1.90 ^c^	0.0001
White	1.65 ^a^	1.88 ^c^	1.76 ^b^	1.88 ^c^	1.91 ^c^	0.0001
Yellow	1.61 ^a^	1.75 ^bc^	1.72 ^b^	1.77 ^bc^	1.79 ^c^	0.0001
Magenta	1.62 ^a^	1.76 ^b^	1.74 ^b^	1.78 ^b^	1.78 ^b^	0.0001
Cyan	1.62 ^a^	1.74 ^bc^	1.70 ^b^	1.78 ^c^	1.75 ^bc^	0.0001
Pink	1.72 ^a^	1.8 9^c^	1.79 ^ab^	1.88 ^c^	1.85 ^bc^	0.0001

Where different letters indicate significantly different values.

**Table 7:** Summary of solutions for soil texture recognition.

Year	Data Type	Processing Technique	No. of Classes	No. of Samples	Replicas	Type of Sample	Acc.	Ref.
2024	Image	CNN	5	392	-	Natural	97.2%	[27]
2019	Image	MIA	6	63	-	Natural	100%	[24]
2022	pXRF, VIS, and NIR data	RF	4	464	-	Natural	97%	[39]
2023	VIS and NIR data	PLSR	-	94	-	Natural	70–90%	[38]
2021	VIS and NIR data	PLSR	-	19	-	Natural	-	[40]
2023	pXRF, VIS, and NIR data	RFE + RF	-	1545	3	Natural	-	[42]
2021	VIS and NIR	PLSR	-	100	-	Natural	-	[41]
2024	VIS data of LDR	DA	5	15	3	Artificial	100%	Proposal

## Data Availability

The data presented in this study are available on request from the corresponding author.
